# An indenide-tethered N-heterocyclic stannylene

**DOI:** 10.1107/S205698902000047X

**Published:** 2020-01-21

**Authors:** Tobias Bischof, Kieren J. Evans, Mairi F. Haddow, Stephen M. Mansell

**Affiliations:** a Institute of Chemical Sciences, School of Engineering and Physical Sciences, Heriot-Watt University, Edinburgh, EH14 4AS, Scotland

**Keywords:** crystal structure, stannylene, η-2 coordination, indenyl donor group

## Abstract

The structure of (μ-1κ*N*:2(η^2^),κ^2^ *N*,*N*′-(2-{[2,6-bis(propan-2-yl)phen­yl]aza­nid­yl}eth­yl)[2-(1*H*-inden-1-yl)eth­yl]aza­nido)(1,4,7,10,13,16-hexa­oxa­cyclo­octa­dec­ane-1κ^6^ *O*)lithiumtin, [LiSn(C_8_H_16_O_4_)(C_25_H_31_N_2_)], at 100 K has monoclinic (*P*2_1_/*n*) symmetry. Analysis of the coordination of the Sn to the indenyl ring shows that the Sn inter­acts in an η^2^ fashion. A database survey showed that whilst this coordination mode is unusual for Ge and Pb compounds, Sn displays a wider range of coordination modes to cyclo­penta­dienyl ligands and their derivatives.

## Abstract 2

Analysis of the coordination of the Sn to the indenyl ring shows that the Sn inter­acts in an η^2^ fashion. A database survey showed that whilst this coordination mode is unusual for Ge and Pb compounds, Sn displays a wider range of coordination modes to cyclo­penta­dienyl ligands and their derivatives.

## Chemical context   

N-heterocyclic stannylenes (NHSns) are the tin analogues of N-heterocyclic carbenes (NHCs). With an unsaturated backbone, they have been found to be thermally unstable (Gans-Eichler *et al.*, 2002 ▸, Gans-Eichler *et al.* 2006 ▸), but with a saturated backbone they are thermally robust (Mansell *et al.*, 2008 ▸) and show inter­esting binding properties including a higher propensity for bridging coordination modes (Mansell *et al.*, 2011 ▸).
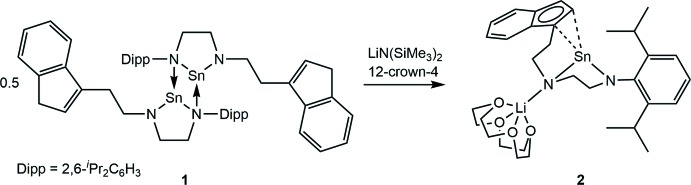


We have sought to install NHSns into a tethered ligand system using a fluorenyl group linked to the NHSn with a C_2_H_4_ linker, but this resulted in dimeric species with Sn—N dative bonding, even upon addition of suitable Rh salts (Roselló-Merino & Mansell, 2016 ▸). In this contribution we analyse the crystal structure of a monomeric NHSn with an indenyl donor group.

## Structural commentary   

**Figure 1:**
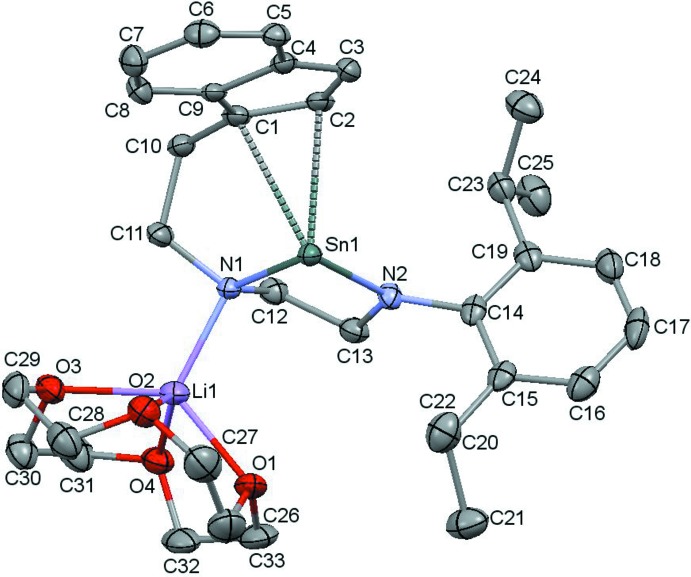
Displacement ellipsoid plot of **2** (shown at the 50% probability level) with all H atoms removed for clarity.

The crystal structure of the title compound **2** shows a deprotonated indenide moiety connected to a di­amido­stannylene unit *via* a C_2_H_4_ linker. The lithium cation is bound to the less sterically hindered N atom [Li—N = 2.043 (7) Å], as well as to the 12-crown-4 tetra­dentate ether ligand (Fig. 1 ▸). The Sn atom is bonded to two N atoms [Sn—N = 2.157 (3) and 2.089 (3) Å] and there appears to be an η^2^ inter­action with the indenyl anion [Sn⋯C = 2.734 (3) and 2.701 (3) Å] with Sn⋯C distances that are similar to those in stannocene [Sn(η^5^-Cp)_2_], Sn⋯C = 2.56 (2)–2.85 (3) Å (Atwood *et al.*, 1981 ▸). The formation of **2** shows that the soft NHSn lone pair does not inter­act with the relatively hard Li cation, unlike the situation in the lithium complexes of tethered NHCs previously published (Evans & Mansell, 2019 ▸; Evans *et al.*, 2019 ▸).

## Database survey   

For the structure of **2**, two Sn⋯C distances are much shorter [2.734 (3) and 2.701 (3) Å] than the other three [3.193 (3), 3.222 (3) and 3.486 (3) Å] in the five-membered ring of the indenyl moiety. The only two other crystallographically characterized Sn-indenyl complexes [Sn{1,3-(SiMe_3_)_2_C_9_H_5_}_2_] and [Sn(C_5_Me_5_){1,3-(SiMe_3_)_2_C_9_H_5_}] (Jones & Cowley, 2005 ▸), have much less pronounced differences in the shortest and longest bond lengths [maximum range of 0.26 Å compared to **2**, which has a range of 0.785 Å] although the bond lengths to two carbon atoms in the ring are shorter than the remaining three, which always includes the two benzannulated carbon atoms. This has been termed η^3^+η^2^ coordination (Calhorda & Veiros, 1999 ▸).

**Figure 2:**
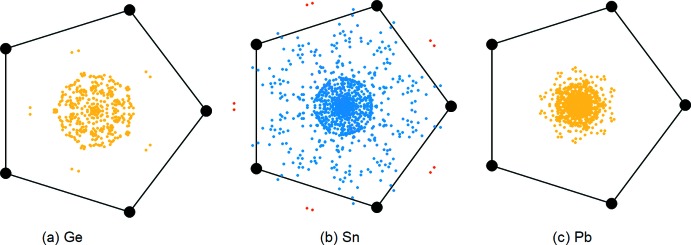
Projection plots of metal position onto idealized Cp ring.

By surveying the coordination of cyclo­penta­dienyl ligands to main group atoms using the CSD (Version 5.40, update of August 2019; Groom *et al.*, 2016 ▸), we can clearly see the flexible coordination modes of tin compared to other group 14 metals. The position of the metal was projected onto the plane of the Cp ring and these datapoints were expanded according to *C*_5v_ symmetry (*i.e*. there are ten symmetry-equivalent data points for each crystal structure). The results are shown in Fig. 2 ▸ *a*–*c* for germanium, tin and lead, respectively. Germanium and lead are almost always projected near the centre of the Cp ring; however, tin shows a wide range of projection points. The datapoints for this structure are displayed in red in Fig. 2 ▸ *b*, showing the distinct inter­action with two carbon centres, a unique coordination mode for group 14 metals.

## Synthesis and crystallization   

**Synthesis of [Sn{(N,N′-κ^2^-(C_9_H_7_)C_2_H_4_NC_2_H_4_N(2,6-** ***^i^*** **Pr_2_C_6_H_3_)}]_2_, 1**

To a solution of (C_9_H_7_)C_2_H_4_N(H)C_2_H_4_N(H)(2,6-^*i*^Pr_2_C_6_H_3_) (Roselló-Merino & Mansell, 2016 ▸) (330 mg, 0.91 mmol) in THF (5 ml), Sn[N(SiMe_3_)_2_]_2_ (400 mg, 0.91 mmol) dissolved in THF (2 ml) was added slowly at room temperature under nitro­gen in a two-necked-flask in a glovebox. After 2 h, the solvent was removed by pipette and the precipitate was washed five times with 5 ml of petroleum ether by dispersing it and pipetting off the solvent after the residue had settled. Evaporation of the remaining solvent under high vacuum yielded the desired product as a light-yellow solid (348 mg, 0.73 mmol, 80%).

^1^H NMR (400 MHz, 298 K, *d* ^8^-THF): δ = 7.5–6.9 (*m*, Ar-H), 6.28 (*m*), 3.67 (*d*), 3.48 (*m*), 3.38 (*m*), 3.00 (*m*), 2.88 (*m*), 2.79 (*m*), 1.19 (*d*); ^119^Sn (149 MHz, 298 K, *d* ^8^-THF); δ = 79.7 ppm. Analysis calculated for C_25_H_32_N_2_Sn: C 62.65, H 6.73, N 5.85; Found: C 62.53, H 6.66, N 5.68

**Synthesis of indenide-tethered N-heterocyclic stannylene 2**

To **1** (10 mg, 0.03 mmol) in a glass vial under nitro­gen in a glovebox was added Li[N(SiMe_3_)_2_] (5 mg, 0.03 mmol) in THF (0.5 mL) then 12-crown-4 (11 mg, 0.6 mmol) in THF (0.2 ml). This vial was placed in a freezer, producing a small number of single crystals. Reactions on larger scales led to concentrations that were too high, leading to decomposition processes. The material that was produced was not soluble in *d* ^8^-THF.

## Refinement   

Crystal data, data collection and structure refinement details are summarized in Table 1 ▸. H atoms were positioned geometrically (C—H = 095–1.00 Å) and refined using a riding model with *U*_iso_(H) = 1.2*U*_eq_(C) or 1.5*U*_eq_(C-meth­yl).

## Supplementary Material

Crystal structure: contains datablock(s) I. DOI: 10.1107/S205698902000047X/ff2165sup1.cif

Supporting information file. DOI: 10.1107/S205698902000047X/ff2165Isup3.cdx

CCDC reference: 1946071

Additional supporting information:  crystallographic information; 3D view; checkCIF report

## Figures and Tables

**Table 1:** Experimental details

Crystal data	
Chemical formula	[LiSn(C_8_H_16_O_4_)(C_25_H_32_N_2_)]
*M*_r_	661.35
Crystal system, space group	Monoclinic, *P*2_1_/*n*
Temperature (K)	100
*a*, *b*, *c* (Å)	9.9766 (8), 17.7991 (14), 18.8402 (14)
β (°)	95.510 (4)
*V* (Å^3^)	3330.1 (4)
*Z*	4
Radiation type	Mo *K*α
μ (mm^−1^)	0.80
Crystal size (mm)	0.20 × 0.20 × 0.08

Data collection	
Diffractometer	Bruker APEXII CCD
Absorption correction	Multi-scan (*SADABS*; Bruker, 2015 ▸)
*T*_min_, *T*_max_	0.614, 0.746
No. of measured, independent and observed [*I* > 2σ(*I*)] reflections	25958, 7615, 5184
*R*_int_	0.051
(sin θ/λ)_max_ (Å^−1^)	0.650

Refinement	
*R*[*F* ^2^ > 2σ(*F* ^2^)], *wR*(*F* ^2^), *S*	0.042, 0.096, 1.04
No. of reflections	7615
No. of parameters	374
H-atom treatment	H-atom parameters constrained
Δρ_max_, Δρ_min_ (e Å^−3^)	1.36, −0.59

Computer programs: *APEX2* and *SAINT* (Bruker, 2013 ▸), *SHELXT* (Sheldrick, 2015*a* ▸), *SHELXL* (Sheldrick, 2015*b* ▸) and *OLEX2* (Dolomanov *et al.*, 2009 ▸).

## supplementary crystallographic information

### Crystal data

**Table:**

[LiSn(C_8_H_16_O_4_)(C_25_H_32_N_2_)]	*F*(000) = 1376
*M**_r_* = 661.35	*D*_x_ = 1.319 Mg m^−^^3^
Monoclinic, *P*2_1_/*n*	Mo *K*α radiation, λ = 0.71073 Å
*a* = 9.9766 (8) Å	Cell parameters from 7023 reflections
*b* = 17.7991 (14) Å	θ = 2.4–25.7°
*c* = 18.8402 (14) Å	µ = 0.80 mm^−^^1^
β = 95.510 (4)°	*T* = 100 K
*V* = 3330.1 (4) Å^3^	Block, pale yellow
*Z* = 4	0.20 × 0.20 × 0.08 mm

### Data collection

**Table:**

Bruker APEXII CCD diffractometer	5184 reflections with *I* > 2σ(*I*)
φ and ω scans	*R*_int_ = 0.051
Absorption correction: multi-scan (SADABS; Bruker, 2015)	θ_max_ = 27.5°, θ_min_ = 2.5°
*T*_min_ = 0.614, *T*_max_ = 0.746	*h* = −12→11
25958 measured reflections	*k* = −23→20
7615 independent reflections	*l* = −24→24

### Refinement

**Table:**

Refinement on *F*^2^	Primary atom site location: structure-invariant direct methods
Least-squares matrix: full	Hydrogen site location: inferred from neighbouring sites
*R*[*F*^2^ > 2σ(*F*^2^)] = 0.042	H-atom parameters constrained
*wR*(*F*^2^) = 0.096	*w* = 1/[σ^2^(*F*_o_^2^) + (0.0367*P*)^2^ + 2.6456*P*] where *P* = (*F*_o_^2^ + 2*F*_c_^2^)/3
*S* = 1.03	(Δ/σ)_max_ = 0.001
7615 reflections	Δρ_max_ = 1.36 e Å^−^^3^
374 parameters	Δρ_min_ = −0.59 e Å^−^^3^
0 restraints	

### Special details

**Table:**

Geometry. All esds (except the esd in the dihedral angle between two l.s. planes) are estimated using the full covariance matrix. The cell esds are taken into account individually in the estimation of esds in distances, angles and torsion angles; correlations between esds in cell parameters are only used when they are defined by crystal symmetry. An approximate (isotropic) treatment of cell esds is used for estimating esds involving l.s. planes.

### Fractional atomic coordinates and isotropic or equivalent isotropic displacement parameters (Å^2^)

**Table:**

	*x*	*y*	*z*	*U*_iso_*/*U*_eq_	
Sn1	0.62831 (2)	0.62730 (2)	0.73575 (2)	0.01853 (8)	
O1	0.7755 (3)	0.69869 (14)	0.93346 (13)	0.0333 (6)	
O2	0.9148 (3)	0.75607 (14)	0.82933 (13)	0.0337 (6)	
O3	0.7698 (3)	0.88333 (13)	0.83378 (12)	0.0291 (6)	
O4	0.6260 (3)	0.82577 (14)	0.93572 (12)	0.0311 (6)	
N1	0.5656 (3)	0.73222 (15)	0.77937 (13)	0.0189 (6)	
N2	0.4936 (3)	0.58853 (15)	0.80578 (14)	0.0208 (6)	
C1	0.5055 (4)	0.70370 (19)	0.62151 (17)	0.0221 (8)	
C2	0.4399 (3)	0.63288 (19)	0.62289 (16)	0.0215 (7)	
H2	0.3654	0.6220	0.6490	0.026*	
C3	0.5018 (4)	0.58133 (19)	0.57967 (17)	0.0226 (8)	
H3	0.4765	0.5303	0.5718	0.027*	
C4	0.6080 (3)	0.61883 (18)	0.55015 (16)	0.0202 (7)	
C5	0.7031 (4)	0.5971 (2)	0.50330 (17)	0.0233 (8)	
H5	0.7044	0.5468	0.4865	0.028*	
C6	0.7938 (4)	0.6485 (2)	0.48200 (19)	0.0293 (9)	
H6	0.8570	0.6335	0.4501	0.035*	
C7	0.7942 (4)	0.7236 (2)	0.50699 (19)	0.0303 (9)	
H7	0.8569	0.7587	0.4913	0.036*	
C8	0.7050 (4)	0.7461 (2)	0.55352 (18)	0.0264 (8)	
H8	0.7068	0.7965	0.5703	0.032*	
C9	0.6111 (4)	0.69545 (18)	0.57669 (17)	0.0198 (8)	
C10	0.4676 (4)	0.77556 (19)	0.65700 (17)	0.0235 (8)	
H10A	0.3728	0.7720	0.6681	0.028*	
H10B	0.4738	0.8177	0.6232	0.028*	
C11	0.5574 (4)	0.79301 (18)	0.72628 (17)	0.0237 (8)	
H11A	0.6495	0.8044	0.7139	0.028*	
H11B	0.5224	0.8387	0.7482	0.028*	
C12	0.4363 (4)	0.72044 (19)	0.80929 (18)	0.0231 (8)	
H12A	0.3623	0.7200	0.7703	0.028*	
H12B	0.4198	0.7622	0.8420	0.028*	
C13	0.4379 (4)	0.64653 (19)	0.84949 (18)	0.0249 (8)	
H13A	0.4937	0.6513	0.8956	0.030*	
H13B	0.3453	0.6328	0.8592	0.030*	
C14	0.4705 (4)	0.51260 (18)	0.82625 (18)	0.0226 (8)	
C15	0.5655 (4)	0.47385 (19)	0.87310 (18)	0.0251 (8)	
C16	0.5425 (4)	0.3981 (2)	0.8873 (2)	0.0331 (9)	
H16	0.6060	0.3714	0.9186	0.040*	
C17	0.4290 (5)	0.3613 (2)	0.8567 (2)	0.0381 (11)	
H17	0.4165	0.3094	0.8658	0.046*	
C18	0.3346 (4)	0.3998 (2)	0.8133 (2)	0.0330 (9)	
H18	0.2560	0.3745	0.7933	0.040*	
C19	0.3522 (4)	0.4757 (2)	0.79801 (18)	0.0250 (8)	
C20	0.6928 (4)	0.5109 (2)	0.90675 (19)	0.0271 (8)	
H20	0.6865	0.5657	0.8952	0.033*	
C21	0.7086 (5)	0.5034 (3)	0.9890 (2)	0.0440 (11)	
H21A	0.6293	0.5247	1.0085	0.066*	
H21B	0.7893	0.5305	1.0084	0.066*	
H21C	0.7172	0.4502	1.0020	0.066*	
C22	0.8186 (4)	0.4806 (2)	0.8764 (2)	0.0392 (10)	
H22A	0.8278	0.4268	0.8871	0.059*	
H22B	0.8980	0.5074	0.8981	0.059*	
H22C	0.8106	0.4880	0.8247	0.059*	
C23	0.2422 (4)	0.5165 (2)	0.75159 (19)	0.0283 (9)	
H23	0.2722	0.5696	0.7462	0.034*	
C24	0.2197 (4)	0.4818 (2)	0.6765 (2)	0.0389 (10)	
H24A	0.3044	0.4828	0.6541	0.058*	
H24B	0.1512	0.5108	0.6475	0.058*	
H24C	0.1893	0.4297	0.6802	0.058*	
C25	0.1108 (4)	0.5182 (2)	0.7863 (2)	0.0414 (11)	
H25A	0.0446	0.5487	0.7573	0.062*	
H25B	0.1266	0.5402	0.8341	0.062*	
H25C	0.0764	0.4669	0.7900	0.062*	
C26	0.9184 (4)	0.6953 (3)	0.9438 (2)	0.0452 (11)	
H26A	0.9476	0.6495	0.9708	0.054*	
H26B	0.9545	0.7397	0.9709	0.054*	
C27	0.9679 (5)	0.6939 (2)	0.8719 (2)	0.0455 (11)	
H27A	1.0675	0.6962	0.8768	0.055*	
H27B	0.9405	0.6461	0.8477	0.055*	
C28	0.9884 (4)	0.8245 (2)	0.8415 (2)	0.0401 (10)	
H28A	1.0747	0.8222	0.8197	0.048*	
H28B	1.0079	0.8338	0.8933	0.048*	
C29	0.9000 (4)	0.8856 (2)	0.8075 (2)	0.0345 (10)	
H29A	0.9423	0.9351	0.8183	0.041*	
H29B	0.8903	0.8789	0.7551	0.041*	
C30	0.7597 (4)	0.9279 (2)	0.8967 (2)	0.0361 (10)	
H30A	0.7581	0.9821	0.8847	0.043*	
H30B	0.8373	0.9181	0.9323	0.043*	
C31	0.6316 (4)	0.9053 (2)	0.9252 (2)	0.0392 (10)	
H31A	0.6239	0.9312	0.9712	0.047*	
H31B	0.5545	0.9211	0.8915	0.047*	
C32	0.6960 (5)	0.8007 (2)	1.00139 (19)	0.0410 (11)	
H32A	0.6439	0.8133	1.0419	0.049*	
H32B	0.7852	0.8253	1.0091	0.049*	
C33	0.7122 (5)	0.7179 (3)	0.9960 (2)	0.0458 (12)	
H33A	0.7675	0.6991	1.0387	0.055*	
H33B	0.6228	0.6934	0.9941	0.055*	
Li1	0.7127 (7)	0.7726 (3)	0.8523 (3)	0.0278 (14)	

### Atomic displacement parameters (Å^2^)

**Table:**

	*U*^11^	*U*^22^	*U*^33^	*U*^12^	*U*^13^	*U*^23^
Sn1	0.02406 (13)	0.01671 (12)	0.01436 (11)	0.00045 (11)	−0.00054 (8)	−0.00026 (10)
O1	0.0362 (17)	0.0370 (15)	0.0244 (14)	−0.0082 (13)	−0.0093 (12)	0.0053 (12)
O2	0.0389 (17)	0.0315 (15)	0.0302 (14)	−0.0023 (13)	0.0007 (13)	−0.0025 (12)
O3	0.0337 (15)	0.0291 (14)	0.0247 (13)	−0.0079 (12)	0.0034 (11)	−0.0044 (11)
O4	0.0347 (16)	0.0370 (16)	0.0213 (13)	−0.0083 (13)	0.0008 (12)	−0.0036 (12)
N1	0.0259 (17)	0.0167 (14)	0.0134 (14)	−0.0006 (12)	−0.0010 (12)	−0.0006 (11)
N2	0.0271 (17)	0.0167 (15)	0.0190 (14)	−0.0017 (12)	0.0050 (13)	−0.0030 (12)
C1	0.025 (2)	0.0235 (18)	0.0161 (17)	0.0013 (16)	−0.0060 (15)	−0.0011 (15)
C2	0.0216 (18)	0.0272 (19)	0.0143 (15)	−0.0012 (16)	−0.0047 (13)	0.0016 (15)
C3	0.030 (2)	0.0181 (18)	0.0182 (17)	−0.0015 (15)	−0.0031 (16)	−0.0018 (14)
C4	0.0253 (19)	0.0199 (18)	0.0145 (15)	0.0028 (16)	−0.0033 (14)	0.0011 (14)
C5	0.029 (2)	0.0232 (18)	0.0164 (17)	0.0020 (16)	−0.0016 (15)	−0.0038 (14)
C6	0.030 (2)	0.038 (2)	0.0213 (19)	0.0017 (17)	0.0049 (16)	−0.0022 (16)
C7	0.030 (2)	0.032 (2)	0.029 (2)	−0.0108 (17)	0.0036 (18)	0.0006 (17)
C8	0.034 (2)	0.0197 (19)	0.0250 (19)	−0.0028 (16)	−0.0008 (17)	−0.0059 (15)
C9	0.026 (2)	0.0186 (17)	0.0131 (16)	0.0013 (15)	−0.0067 (14)	−0.0005 (13)
C10	0.031 (2)	0.0213 (18)	0.0168 (17)	0.0023 (16)	−0.0039 (15)	0.0013 (14)
C11	0.033 (2)	0.0183 (18)	0.0184 (17)	0.0011 (16)	−0.0029 (16)	−0.0046 (14)
C12	0.026 (2)	0.0223 (19)	0.0206 (18)	0.0024 (16)	0.0031 (16)	−0.0038 (15)
C13	0.028 (2)	0.027 (2)	0.0199 (17)	−0.0024 (16)	−0.0001 (15)	−0.0042 (15)
C14	0.033 (2)	0.0181 (18)	0.0190 (17)	0.0004 (16)	0.0139 (16)	−0.0018 (14)
C15	0.033 (2)	0.0246 (19)	0.0195 (18)	0.0022 (17)	0.0120 (16)	0.0016 (15)
C16	0.040 (3)	0.029 (2)	0.032 (2)	0.0072 (19)	0.0117 (19)	0.0048 (17)
C17	0.056 (3)	0.023 (2)	0.039 (2)	−0.007 (2)	0.025 (2)	0.0043 (18)
C18	0.039 (3)	0.029 (2)	0.032 (2)	−0.0115 (18)	0.0089 (19)	−0.0018 (17)
C19	0.029 (2)	0.0254 (19)	0.0222 (18)	−0.0050 (16)	0.0121 (16)	−0.0064 (15)
C20	0.030 (2)	0.027 (2)	0.0251 (19)	0.0017 (17)	0.0044 (16)	0.0071 (16)
C21	0.043 (3)	0.061 (3)	0.028 (2)	−0.002 (2)	0.002 (2)	0.007 (2)
C22	0.039 (3)	0.037 (2)	0.044 (3)	0.005 (2)	0.012 (2)	0.007 (2)
C23	0.029 (2)	0.025 (2)	0.031 (2)	−0.0057 (16)	0.0043 (18)	−0.0043 (15)
C24	0.042 (3)	0.044 (3)	0.031 (2)	−0.005 (2)	0.0024 (19)	−0.0105 (19)
C25	0.040 (3)	0.039 (2)	0.047 (3)	−0.006 (2)	0.012 (2)	−0.009 (2)
C26	0.041 (3)	0.047 (3)	0.044 (3)	−0.003 (2)	−0.012 (2)	0.011 (2)
C27	0.043 (3)	0.038 (2)	0.053 (3)	0.006 (2)	−0.008 (2)	−0.001 (2)
C28	0.036 (3)	0.042 (3)	0.043 (2)	−0.011 (2)	0.007 (2)	−0.007 (2)
C29	0.040 (2)	0.031 (2)	0.034 (2)	−0.0124 (19)	0.0101 (19)	0.0029 (18)
C30	0.045 (3)	0.029 (2)	0.035 (2)	−0.0048 (19)	0.010 (2)	−0.0086 (18)
C31	0.044 (3)	0.034 (2)	0.040 (2)	−0.002 (2)	0.008 (2)	−0.0114 (19)
C32	0.048 (3)	0.059 (3)	0.0156 (19)	−0.016 (2)	0.0013 (18)	−0.0004 (19)
C33	0.057 (3)	0.061 (3)	0.017 (2)	−0.014 (2)	−0.004 (2)	0.006 (2)
Li1	0.038 (4)	0.028 (3)	0.017 (3)	−0.005 (3)	−0.004 (3)	0.000 (3)

### Geometric parameters (Å, º)

**Table:**

Sn1—N1	2.157 (3)	C15—C16	1.398 (5)
Sn1—N2	2.089 (3)	C15—C20	1.515 (5)
O1—C26	1.422 (5)	C16—H16	0.9500
O1—C33	1.430 (5)	C16—C17	1.386 (6)
O1—Li1	2.068 (6)	C17—H17	0.9500
O2—C27	1.438 (5)	C17—C18	1.371 (6)
O2—C28	1.430 (5)	C18—H18	0.9500
O2—Li1	2.123 (7)	C18—C19	1.395 (5)
O3—C29	1.435 (4)	C19—C23	1.521 (5)
O3—C30	1.437 (4)	C20—H20	1.0000
O3—Li1	2.090 (6)	C20—C21	1.548 (5)
O4—C31	1.431 (5)	C20—C22	1.526 (5)
O4—C32	1.432 (4)	C21—H21A	0.9800
O4—Li1	2.091 (7)	C21—H21B	0.9800
N1—C11	1.470 (4)	C21—H21C	0.9800
N1—C12	1.471 (4)	C22—H22A	0.9800
N1—Li1	2.043 (7)	C22—H22B	0.9800
N2—C13	1.463 (4)	C22—H22C	0.9800
N2—C14	1.430 (4)	C23—H23	1.0000
C1—C2	1.422 (5)	C23—C24	1.540 (5)
C1—C9	1.420 (5)	C23—C25	1.521 (5)
C1—C10	1.508 (5)	C24—H24A	0.9800
C2—H2	0.9500	C24—H24B	0.9800
C2—C3	1.408 (5)	C24—H24C	0.9800
C3—H3	0.9500	C25—H25A	0.9800
C3—C4	1.411 (5)	C25—H25B	0.9800
C4—C5	1.411 (5)	C25—H25C	0.9800
C4—C9	1.452 (4)	C26—H26A	0.9900
C5—H5	0.9500	C26—H26B	0.9900
C5—C6	1.374 (5)	C26—C27	1.486 (6)
C6—H6	0.9500	C27—H27A	0.9900
C6—C7	1.418 (5)	C27—H27B	0.9900
C7—H7	0.9500	C28—H28A	0.9900
C7—C8	1.367 (5)	C28—H28B	0.9900
C8—H8	0.9500	C28—C29	1.504 (6)
C8—C9	1.400 (5)	C29—H29A	0.9900
C10—H10A	0.9900	C29—H29B	0.9900
C10—H10B	0.9900	C30—H30A	0.9900
C10—C11	1.542 (5)	C30—H30B	0.9900
C11—H11A	0.9900	C30—C31	1.489 (5)
C11—H11B	0.9900	C31—H31A	0.9900
C12—H12A	0.9900	C31—H31B	0.9900
C12—H12B	0.9900	C32—H32A	0.9900
C12—C13	1.517 (5)	C32—H32B	0.9900
C13—H13A	0.9900	C32—C33	1.487 (6)
C13—H13B	0.9900	C33—H33A	0.9900
C14—C15	1.412 (5)	C33—H33B	0.9900
C14—C19	1.410 (5)		
			
N2—Sn1—N1	79.49 (10)	C15—C20—C22	112.1 (3)
C26—O1—C33	114.4 (3)	C21—C20—H20	107.5
C26—O1—Li1	110.9 (3)	C22—C20—H20	107.5
C33—O1—Li1	109.3 (3)	C22—C20—C21	109.6 (3)
C27—O2—Li1	107.4 (3)	C20—C21—H21A	109.5
C28—O2—C27	114.2 (3)	C20—C21—H21B	109.5
C28—O2—Li1	109.4 (3)	C20—C21—H21C	109.5
C29—O3—C30	114.0 (3)	H21A—C21—H21B	109.5
C29—O3—Li1	110.8 (3)	H21A—C21—H21C	109.5
C30—O3—Li1	109.9 (3)	H21B—C21—H21C	109.5
C31—O4—C32	113.9 (3)	C20—C22—H22A	109.5
C31—O4—Li1	108.6 (3)	C20—C22—H22B	109.5
C32—O4—Li1	107.8 (3)	C20—C22—H22C	109.5
C11—N1—Sn1	112.15 (19)	H22A—C22—H22B	109.5
C11—N1—C12	111.9 (3)	H22A—C22—H22C	109.5
C11—N1—Li1	100.8 (3)	H22B—C22—H22C	109.5
C12—N1—Sn1	108.64 (19)	C19—C23—H23	107.7
C12—N1—Li1	113.1 (3)	C19—C23—C24	111.7 (3)
Li1—N1—Sn1	110.2 (2)	C19—C23—C25	111.5 (3)
C13—N2—Sn1	115.1 (2)	C24—C23—H23	107.7
C14—N2—Sn1	127.8 (2)	C25—C23—H23	107.7
C14—N2—C13	115.8 (3)	C25—C23—C24	110.4 (3)
C2—C1—C10	127.4 (3)	C23—C24—H24A	109.5
C9—C1—C2	106.8 (3)	C23—C24—H24B	109.5
C9—C1—C10	125.7 (3)	C23—C24—H24C	109.5
C1—C2—H2	125.1	H24A—C24—H24B	109.5
C3—C2—C1	109.8 (3)	H24A—C24—H24C	109.5
C3—C2—H2	125.1	H24B—C24—H24C	109.5
C2—C3—H3	126.0	C23—C25—H25A	109.5
C2—C3—C4	107.9 (3)	C23—C25—H25B	109.5
C4—C3—H3	126.0	C23—C25—H25C	109.5
C3—C4—C9	107.5 (3)	H25A—C25—H25B	109.5
C5—C4—C3	133.8 (3)	H25A—C25—H25C	109.5
C5—C4—C9	118.7 (3)	H25B—C25—H25C	109.5
C4—C5—H5	119.9	O1—C26—H26A	110.3
C6—C5—C4	120.1 (3)	O1—C26—H26B	110.3
C6—C5—H5	119.9	O1—C26—C27	107.0 (3)
C5—C6—H6	119.6	H26A—C26—H26B	108.6
C5—C6—C7	120.8 (3)	C27—C26—H26A	110.3
C7—C6—H6	119.6	C27—C26—H26B	110.3
C6—C7—H7	119.8	O2—C27—C26	111.0 (4)
C8—C7—C6	120.5 (3)	O2—C27—H27A	109.4
C8—C7—H7	119.8	O2—C27—H27B	109.4
C7—C8—H8	119.7	C26—C27—H27A	109.4
C7—C8—C9	120.5 (3)	C26—C27—H27B	109.4
C9—C8—H8	119.7	H27A—C27—H27B	108.0
C1—C9—C4	108.0 (3)	O2—C28—H28A	110.5
C8—C9—C1	132.5 (3)	O2—C28—H28B	110.5
C8—C9—C4	119.4 (3)	O2—C28—C29	106.0 (3)
C1—C10—H10A	108.9	H28A—C28—H28B	108.7
C1—C10—H10B	108.9	C29—C28—H28A	110.5
C1—C10—C11	113.3 (3)	C29—C28—H28B	110.5
H10A—C10—H10B	107.7	O3—C29—C28	110.2 (3)
C11—C10—H10A	108.9	O3—C29—H29A	109.6
C11—C10—H10B	108.9	O3—C29—H29B	109.6
N1—C11—C10	114.7 (3)	C28—C29—H29A	109.6
N1—C11—H11A	108.6	C28—C29—H29B	109.6
N1—C11—H11B	108.6	H29A—C29—H29B	108.1
C10—C11—H11A	108.6	O3—C30—H30A	110.5
C10—C11—H11B	108.6	O3—C30—H30B	110.5
H11A—C11—H11B	107.6	O3—C30—C31	106.2 (3)
N1—C12—H12A	109.6	H30A—C30—H30B	108.7
N1—C12—H12B	109.6	C31—C30—H30A	110.5
N1—C12—C13	110.3 (3)	C31—C30—H30B	110.5
H12A—C12—H12B	108.1	O4—C31—C30	111.3 (3)
C13—C12—H12A	109.6	O4—C31—H31A	109.4
C13—C12—H12B	109.6	O4—C31—H31B	109.4
N2—C13—C12	108.5 (3)	C30—C31—H31A	109.4
N2—C13—H13A	110.0	C30—C31—H31B	109.4
N2—C13—H13B	110.0	H31A—C31—H31B	108.0
C12—C13—H13A	110.0	O4—C32—H32A	110.3
C12—C13—H13B	110.0	O4—C32—H32B	110.3
H13A—C13—H13B	108.4	O4—C32—C33	107.3 (3)
C15—C14—N2	121.0 (3)	H32A—C32—H32B	108.5
C19—C14—N2	119.3 (3)	C33—C32—H32A	110.3
C19—C14—C15	119.7 (3)	C33—C32—H32B	110.3
C14—C15—C20	122.3 (3)	O1—C33—C32	110.6 (3)
C16—C15—C14	118.6 (4)	O1—C33—H33A	109.5
C16—C15—C20	119.1 (3)	O1—C33—H33B	109.5
C15—C16—H16	119.4	C32—C33—H33A	109.5
C17—C16—C15	121.3 (4)	C32—C33—H33B	109.5
C17—C16—H16	119.4	H33A—C33—H33B	108.1
C16—C17—H17	120.1	O1—Li1—O2	80.6 (2)
C18—C17—C16	119.9 (4)	O1—Li1—O3	131.0 (3)
C18—C17—H17	120.1	O1—Li1—O4	81.4 (2)
C17—C18—H18	119.5	O3—Li1—O2	79.5 (2)
C17—C18—C19	121.0 (4)	O3—Li1—O4	80.7 (2)
C19—C18—H18	119.5	O4—Li1—O2	133.3 (3)
C14—C19—C23	121.7 (3)	N1—Li1—O1	114.9 (3)
C18—C19—C14	119.4 (4)	N1—Li1—O2	116.7 (3)
C18—C19—C23	118.9 (3)	N1—Li1—O3	114.1 (3)
C15—C20—H20	107.5	N1—Li1—O4	110.0 (3)
C15—C20—C21	112.4 (3)		
			
Sn1—N1—C11—C10	54.6 (3)	C13—N2—C14—C15	−92.8 (4)
Sn1—N1—C12—C13	41.9 (3)	C13—N2—C14—C19	88.4 (4)
Sn1—N2—C13—C12	27.7 (3)	C14—N2—C13—C12	−164.2 (3)
Sn1—N2—C14—C15	73.5 (4)	C14—C15—C16—C17	−0.4 (5)
Sn1—N2—C14—C19	−105.3 (3)	C14—C15—C20—C21	126.3 (4)
O1—C26—C27—O2	54.0 (5)	C14—C15—C20—C22	−109.8 (4)
O2—C28—C29—O3	53.8 (4)	C14—C19—C23—C24	118.9 (4)
O3—C30—C31—O4	54.0 (4)	C14—C19—C23—C25	−117.1 (4)
O4—C32—C33—O1	54.1 (5)	C15—C14—C19—C18	−4.0 (5)
N1—C12—C13—N2	−45.8 (4)	C15—C14—C19—C23	175.6 (3)
N2—C14—C15—C16	−175.5 (3)	C15—C16—C17—C18	−2.0 (6)
N2—C14—C15—C20	2.8 (5)	C16—C15—C20—C21	−55.4 (4)
N2—C14—C19—C18	174.8 (3)	C16—C15—C20—C22	68.5 (4)
N2—C14—C19—C23	−5.6 (5)	C16—C17—C18—C19	1.3 (6)
C1—C2—C3—C4	0.0 (4)	C17—C18—C19—C14	1.7 (5)
C1—C10—C11—N1	−54.3 (4)	C17—C18—C19—C23	−177.9 (3)
C2—C1—C9—C4	0.6 (4)	C18—C19—C23—C24	−61.6 (4)
C2—C1—C9—C8	177.3 (4)	C18—C19—C23—C25	62.4 (4)
C2—C1—C10—C11	102.2 (4)	C19—C14—C15—C16	3.3 (5)
C2—C3—C4—C5	−179.4 (3)	C19—C14—C15—C20	−178.4 (3)
C2—C3—C4—C9	0.4 (4)	C20—C15—C16—C17	−178.7 (3)
C3—C4—C5—C6	177.9 (4)	C26—O1—C33—C32	90.1 (4)
C3—C4—C9—C1	−0.6 (4)	C27—O2—C28—C29	−166.0 (3)
C3—C4—C9—C8	−177.8 (3)	C28—O2—C27—C26	83.4 (4)
C4—C5—C6—C7	0.5 (5)	C29—O3—C30—C31	−167.3 (3)
C5—C4—C9—C1	179.2 (3)	C30—O3—C29—C28	89.4 (4)
C5—C4—C9—C8	2.0 (5)	C31—O4—C32—C33	−165.3 (3)
C5—C6—C7—C8	0.8 (6)	C32—O4—C31—C30	82.2 (4)
C6—C7—C8—C9	−0.6 (6)	C33—O1—C26—C27	−166.2 (3)
C7—C8—C9—C1	−177.1 (4)	Li1—O1—C26—C27	−42.0 (4)
C7—C8—C9—C4	−0.8 (5)	Li1—O1—C33—C32	−35.0 (4)
C9—C1—C2—C3	−0.4 (4)	Li1—O2—C27—C26	−38.1 (4)
C9—C1—C10—C11	−81.5 (4)	Li1—O2—C28—C29	−45.6 (4)
C9—C4—C5—C6	−1.9 (5)	Li1—O3—C29—C28	−35.1 (4)
C10—C1—C2—C3	176.5 (3)	Li1—O3—C30—C31	−42.3 (4)
C10—C1—C9—C4	−176.4 (3)	Li1—O4—C31—C30	−37.9 (4)
C10—C1—C9—C8	0.3 (6)	Li1—O4—C32—C33	−44.7 (4)
C11—N1—C12—C13	166.3 (3)	Li1—N1—C11—C10	171.8 (3)
C12—N1—C11—C10	−67.8 (4)	Li1—N1—C12—C13	−80.7 (3)
